# Reporting guidelines should be free to publish, read, and use

**DOI:** 10.7189/jogh.10.0203107

**Published:** 2020-12

**Authors:** Patricia Logullo, Jennifer A de Beyer, Shona Kirtley, Caroline Struthers, Gary S Collins

**Affiliations:** 1 UK EQUATOR Centre, Centre for Statistics in Medicine, Nuffield Department of Orthopaedics, Rheumatology & Musculoskeletal Sciences, University of Oxford, Oxford, United Kingdom; 2 National Institute for Health Research Oxford Biomedical Research Centre, John Radcliffe Hospital, Oxford, United Kingdom

Reporting guidelines are important tools aimed at improving transparency and reproducibility in health research [1-6]. However, many reporting guidelines are currently hidden behind paywalls and restrictive copyright licences, which is in direct opposition to their stated goal. The UK EQUATOR Centre (https://www.equator-network.org/about-us/uk-equator-centre/) argues that tools created to improve transparency in research should be freely accessible for all researchers to read, share, use, and build upon and that publishers should facilitate this by waiving article processing charges (APCs) from reporting guidelines.

Reporting guidelines reduce research waste by helping authors write a complete account of their study, allowing others, in theory, to reproduce it. They prompt authors to include the information needed for readers to understand what was done, how, why, and the implications of the results [1,2]. They are usually published as papers in scientific journals. The most used and updated reporting guidelines are published in a short “statement” paper describing their development, with a checklist of the minimum set of items to be reported. These checklist papers are often accompanied by a longer explanation and elaboration (E&E) paper, usually a separate publication explaining each item and providing examples of complete reporting [6].

Despite over a decade of promoting reporting guidelines by the EQUATOR Network [3], International Committee for Medical Journal Editors [4], publishers, journals [7], the Committee of Publication Ethics [8], and others, incomplete and inadequate reports of health research studies continue to be published [9,10]. One reason may be that authors are unable to access reporting guidelines, their associated checklists, or the helpful explanations and examples of good reporting in the E&E papers.

## COPYRIGHT LICENCE CHOICES CAN RESTRICT GUIDELINE USE

Journals that require a checklist at submission from authors often link to appropriate reporting guidelines or the EQUATOR Network in their instructions for authors. They, and many visitors to the EQUATOR website, assume that everyone will be able to access the guidelines, checklists, and associated E&E papers. However, this is not the case. Reporting guideline papers are all subject to some level of copyright restriction, depending on where and how they are published.

**Figure:**
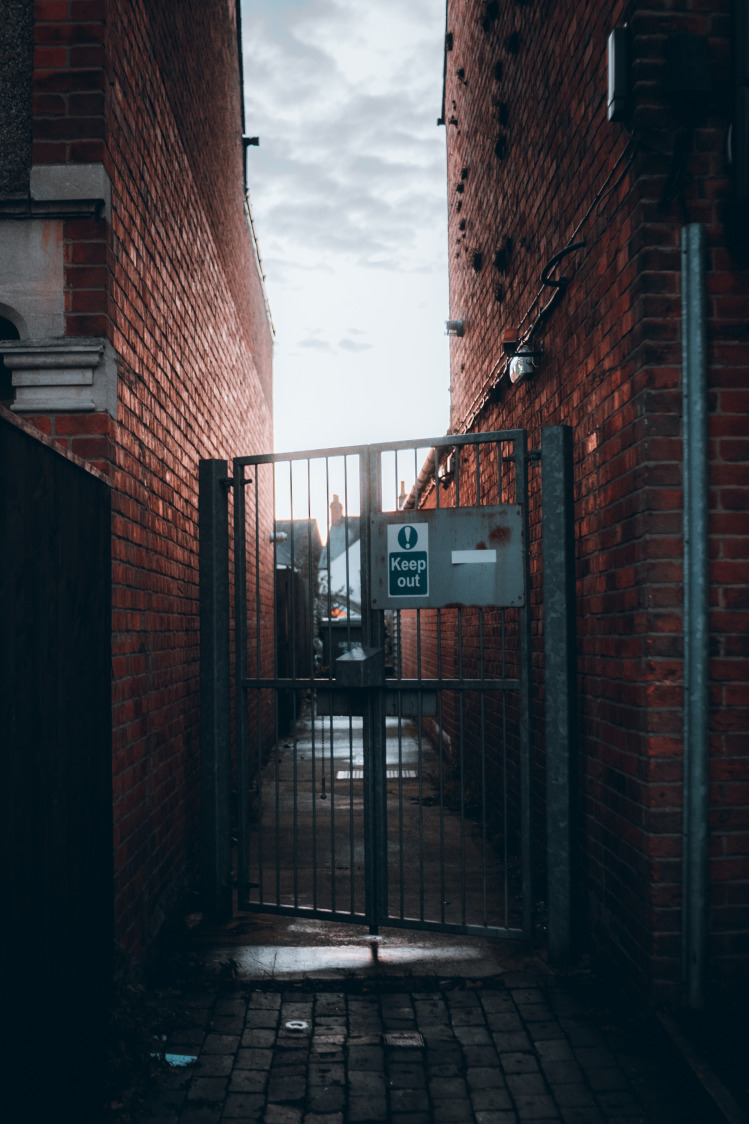
Photo: Restrictive copyright licences make it difficult to use of reporting guidelines. Credit to Matheus Bardemaker on Flickr, CCBY.

In traditional publishing, journals recoup their expenses by charging readers for access. Researchers that cannot rely on university or organisational subscriptions to access journals must instead pay individual pay-to-access fees. Locking reporting guidelines – tools designed for everyone to use – behind such paywalls disadvantages potential guideline users who are not affiliated with academic institutions or who are citizens of low- and middle-income countries or organisations that do not have the needed journal subscriptions.

To make the article immediately free to read, many authors of reporting guidelines have turned to gold open access, paying an upfront article processing charge (APC). Alternatively, some subscription journals make an article free to read some time after publication (green open access). “Free to read” is a good first step: researchers can access and read the reporting guideline and E&E paper and use them as a guide in writing their own articles and peer-reviewing others.

However, free to read does not mean free to use. As with all written work, the text, figures, and tables in a reporting guideline paper are automatically under copyright. Researchers must seek permission from the copyright holder to share (using any media), create copies, publish (for example, on a website), or use the item to create other works. The ability to access and read an article does not imply permission for use; this must be explicitly granted. Strictly speaking, if a reporting guideline is under a restrictive copyright licence, a researcher cannot copy its checklist and submit it to a journal alongside their manuscript, unless they have been granted permission from the copyright holder, usually the publisher. It is impractical to require every researcher to request permission each time they wish to submit a checklist, particularly if they need to pay for the privilege!

The Creative Commons initiative provides six widely used open-access licences that waive certain sets of rights. The choice of open-access licence should be made carefully to avoid restricting desired activities. The Creative Commons Attribution (CC BY) licence allows users to “distribute, remix, adapt, and build upon your work, even commercially, as long as they credit you for the original creation” [11]. Other licences are more restrictive, for instance only allowing non-commercial uses (CC BY-NC) [12] or not allowing any derivative works (CC BY-ND) [13]. Some of these licences would make it difficult to approve translations of reporting guidelines, for example.

A restrictive licence can also prevent other researchers from developing ways to make reporting guidelines more accessible and user-friendly [14] by putting them into open-access online databases or using them to create article templates, online decision tools, or interactive checklists. The UK EQUATOR Centre has encountered this problem in developing our award-winning GoodReports online checklist and template tool for all types of health research [15,16]. Many of the most important reporting guidelines identified for inclusion in GoodReports – or their E&E papers – are copyright-restricted. Some publishers have been reluctant to waive or reduce their reuse fees, which are often determined by the potential number of users or are annual fees for unlimited use. This position is unhelpful and not aligned with the ethos underpinning reporting guidelines. It is a barrier for important and much-needed initiatives to help improve published health research.

## PUBLISH ALL REPORTING GUIDANCE FULLY OPEN ACCESS, WITHOUT CHARGING APCS

In our experience at the EQUATOR Network, developers of reporting guidelines are often surprised to discover that their published guideline cannot be used as fully as they had hoped. We recommend that reporting guideline developers make sure that all reporting guidance – the checklist, the paper it is introduced in, and any accompanying E&E paper – will be freely accessible to all users [6].

Although they contribute to tackling research waste, many reporting guidelines are unfunded. It is therefore unreasonable to ask authors to pay as much as £4000 to publish two papers for educational purposes, particularly if we are to encourage researchers from less well-off institutions to join development groups. We call on publishers and journals to waive APCs for reporting guidance. They have much to gain from publishing these papers under a CC BY licence: checklists and E&E papers receive thousands of citations each year [17] and show a publisher’s commitment to research transparency and reproducibility.

If after publication, reporting guideline authors find that their original papers are more restricted than they had intended, we encourage them to negotiate with their publisher that they be allowed to release at least their checklist under a CC BY licence, if not the entire paper and E&E. The checklists can then be made available on dedicated websites or through the EQUATOR Network and can be freely submitted to journals as part of the manuscript submission process.

Reporting guideline updates are an excellent opportunity to switch to a more appropriate licence to enable full use of these transparency tools. Reporting guideline development groups can also explicitly require that all E&Es, extensions, updates, and translations associated with their guideline be appropriately licenced.

To determine the extent of the use of restrictive licences and better understand the reporting guideline landscape more generally, the UK EQUATOR Centre is undertaking a comprehensive evaluation of its database of over 400 reporting guidelines for health research [18]. This baseline information will help inform efforts to increase the proportion of reporting guidelines that are published fully open access and open use.

## CONCLUSION

Developing a high-quality consensus-based reporting guideline is a time-consuming and rigorous process. It is regrettable that many guidelines developed to help authors report their studies cannot be used to their full potential simply because the reporting guideline was not published in an appropriate open-access format with a licence that allows it to be used. The UK EQUATOR Centre urges publishers, journals, funders, and developers to ensure that all reporting guidelines, adhering to strict quality standards, are published fully open access, using a CC BY licence, and to waive APCs. This model will lead to greater awareness and appreciation of the importance of reporting guidelines as educational tools that can be used and adapted to promote research integrity and to deliver much-needed improvements in science reporting.
